# sCMOS-based fNIRS system: validation via optical performance and cortical response

**DOI:** 10.1038/s41398-026-03992-w

**Published:** 2026-04-09

**Authors:** Jie Zhou, Bingzi Yan, Yang Pu, Lin Huang, Yan Luo

**Affiliations:** 1 Ultrasound department of West China Hospital of Sichuan University, Chengdu, Sichuan 610041 China; 2 School of Electronic Science and Engineering, University of Electronic Science and Technology, Chengdu, Sichuan 611731 China

**Keywords:** Neuroscience, Psychiatric disorders

## Abstract

Functional near-infrared spectroscopy (fNIRS) is a non-invasive neuroimaging technique for assessing cerebral function, combining excellent ecological validity with high temporal resolution. However, despite the advantages of fNIRS, challenges remain including insufficient spatial resolution, and bulkiness—issues that conventional fNIRS devices inadequately address, particularly given the notable increase in equipment bulk and costs associated with enhancing resolution by increasing the number of detectors. In this study, we propose a scientific complementary metal-oxide-semiconductor (sCMOS) sensor as a viable alternative to fNIRS detectors. The sCMOS sensor offers a two-dimensional array sensor that replaces the traditional configuration of multiple diode-lock-in amplifiers, significantly reducing both the size and cost of the devices. However, this raises a crucial question: can the sCMOS sensor effectively substitute traditional detectors while maintaining comparable accuracy to the weak scattered light characteristic of fNIRS? To address this question, we conducted comprehensive validation through both in vitro optical performance (including scattering-absorption and blood-doped tissue-simulating phantoms) and in vivo cortical response. Our results demonstrate that the developed sCMOS-based fNIRS system achieves detection performance comparable to conventional fNIRS technologies, indicating strong potential for translational applications.

## Introduction

In 1977, Frans Jöbsis directed near-infrared (NIR) light unilaterally into the temporal region and detected emitted photons on the contralateral side [1]. Subsequently, he successfully captured the initial signals in NIR spectroscopic cerebral functional signals, thereby establishing the fundamental groundwork for functional near-infrared spectroscopy (fNIRS) technology.

Conceptually, fNIRS involves transmitting NIR light into tissues and measuring backscattered light intensity. Hemoglobin chromophores exhibit characteristic NIR absorption spectra that vary with their oxygenation state. Based on these optical properties, fNIRS detects changes in the concentrations of oxygenated hemoglobin (ΔHbO) and deoxygenated hemoglobin (ΔHbR): neuronal activity triggers region-specific cerebral blood flow increases within seconds [2], resulting in measurable ΔHbO and ΔHbR, a phenomenon known as the hemodynamic response.

fNIRS is a non-invasive, motion-tolerant, and cost-effective neuroimaging tool for assessing cerebral hemodynamics, offering a practical alternative to functional magnetic resonance imaging (fMRI) [3]. Its portability and compatibility with naturalistic settings make it ideal for studying neurodevelopmental and clinical populations, including pediatric, psychiatric, and neurological cohorts [3, 4]. Additionally, fNIRS demonstrates strong electromagnetic compatibility [5], enabling multimodal integration with electroencephalography (EEG) [6], fMRI [7], as well as neural stimulation [8, 9].

Over the course of its development, fNIRS instrumentation has evolved from single-channel measurement systems using a pair of source-detector optodes to high-density systems comprising hundreds of channels covering the entire head. However, a recent review of fNIRS technology development noted that, although device architectures are advancing toward enhanced portability, increased channel density, and wireless functionality, the attainable field-of-view remains inherently limited by the number of source-detector optodes that can be physically deployed (or the density of modular components) [10]. This fundamental physical constraint presents a continuing challenge to the scalability of next-generation high-density and modular systems.

When fNIRS technology was initially developed in Western countries, the majority of subjects possessed lighter hair and skin tones. Consequently, during this period, photodiode (PD) technology was commonly used for detectors. However, darker hair colors and skin tones significantly impact light absorption [11, 12], making it challenging to obtain effective detection signals using PD technology in populations beyond those of Caucasian descent. In contrast to PD, avalanche photodiode (APD) offers higher gain and improved signal-to-noise ratio, rendering it the most frequently utilized photoelectric conversion device in fNIRS applications [13]. The APD operates by leveraging the impact ionization process, wherein photo-generated carriers are continuously accelerated by a strong electric field to generate new electron-hole pairs, ultimately leading to an avalanche effect. This process enables the device to produce substantial current even under low light intensities. Compared to PD, APD offers advantages such as lower noise, higher sensitivity, faster response time, and longer operational lifespan, although it may cost several times more to manufacture [13]. Furthermore, even more expensive yet more sensitive photon sensors are available, including the single-photon avalanche diode, photomultiplier tube (PMT), and micro-channel plate PMT. While the aforementioned detectors can elevate low-light sensing to an unprecedented level, they offer no advantages in terms of cost or instrument size. The challenge does not stem from the detectors themselves, but rather from the design of single-channel detection. Implementing a unified detector array capable of multichannel acquisition could simultaneously address the volumetric inefficiency of conventional fNIRS systems and potentially reduce production costs.

In 2018, Bergonzi et al. reported a scientific complementary metal-oxide-semiconductor (sCMOS) sensor-based high-density diffuse optical tomography design. They highlighted the advantages of sCMOS cameras, including their lower cost alongside improving performance [14]. However, the concurrent requirements for portability and robust optical coupling present a fundamental paradox in fNIRS instrumentation [15]. This raises critical questions regarding the technical viability of sCMOS detectors as replacements for conventional fNIRS: Can sCMOS-based fNIRS achieve comparable accuracy to traditional systems in detecting the weak scattered light signals?

Therefore, this study utilized an sCMOS detector for fNIRS devices. The sCMOS-based fNIRS platform incorporates a power source, laser diode (LD) light sources, an sCMOS, optical fibers, a head-mounted assembly, and a control computer. We performed comprehensive characterization of this prototype to assess its in vitro optical performance and its in vivo cortical response, comparing its performance to that of APD-based fNIRS systems. The results demonstrated that the sCMOS-based fNIRS system achieved photodetection accuracy comparable to APD-based systems. Additionally, it offered the advantage of a smaller size, making it suitable for portable fNIRS. The validated sCMOS-based fNIRS holds substantial translational potential for psychiatric neuroimaging.

## Subjects and methods

### sCMOS-based fNIRS system

**Fig. 1:**
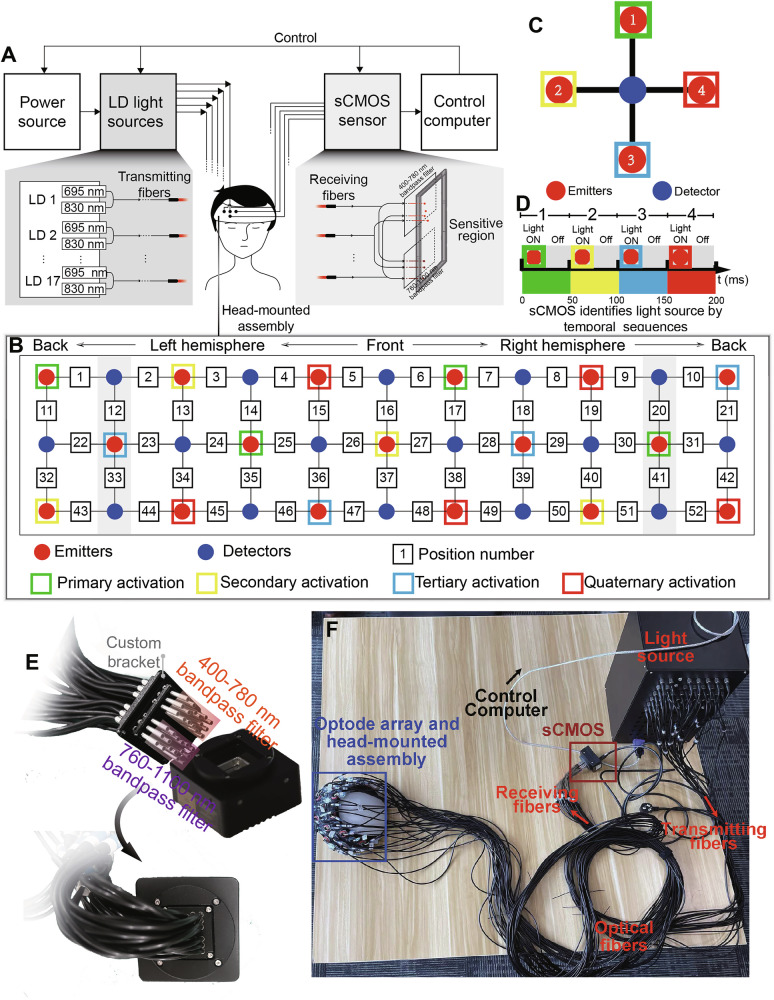
sCMOS-based fNIRS system. **A** System overview comprising a power source, LD sources (690/830 nm), transmitting/receiving fibers, an sCMOS sensor, a head-mounted assembly, and a control computer. **B** 3 × 11 optode array with sequentially triggered emitters (green→yellow→blue→red) under cyclic control. **C** Single-detector activation protocol. **D** The sCMOS-based system identifies light sources by their temporal sequences. **E** Received light is spectrally filtered (400–780 nm / 760–1100 nm bandpass) before detection by the sCMOS sensor. A custom bracket fixes the fiber-optic termini while providing light shielding. **F** The integrated hardware configuration.

The sCMOS-based fNIRS hardware (Fig. 1A) comprises a power source, LD light sources, an sCMOS sensor, optical fibers (including transmitting and receiving fibers), a head-mounted assembly, and a control computer.

Specifically, the light sources consisted of 34 NIR LDs, with 17 emitting wavelength of 695 nm and the remaining 17 at 830 nm. These wavelengths were chosen on the basis of established optophysiological principles to optimize tissue penetration and hemoglobin sensitivity [16]. The light output was calibrated to approximately 4 mW at the tip of the transmitting fiber, which has a diameter of 3 mm, resulting in an irradiance of 56.6 mW/cm² per wavelength. Light at the two wavelengths is combined into a single transmission fiber, the tip of which serves as the emitter. For safety, the total optical irradiance (after combining both wavelengths) and the average power comply with the laser safety standards established by the People’s Republic of China (GB 7247.1-2012) and the American National Standards Institute (ANSI Z136.1-2007). The output is below the maximum permissible exposure (MPE) for skin, as defined by both standards: 200 mW/cm^2^ (695 nm) and 363.94 mW/cm^2^ (830 nm) across both regulatory frameworks.

The optode array was arranged in a 3 × 11 grid, comprising 17 light sources and 16 detectors, which formed a total of 52 measurement channels [17] (Fig. 1B). The source-detector separation (SDS) was set to 30 mm. Each emitter was typically surrounded by four detectors, and each detector by four emitters; this number was reduced to two or three at the array edges due to boundary effects.

The light sources were divided into four groups (Fig. 1B-D, green, yellow, blue, and red frames), which were activated sequentially by a trigger signal in a repeating cycle. The activation timing for a representative detector surrounded by four sources is detailed in Fig. 1C. Each source emitted light for a 25-ms duration, followed by a 25-ms off period. The light was delivered via transmitting fibers to irradiate the scalp and underlying cortex. After undergoing reflection and scattering within the tissue, a portion of the light was collected by the receiving fibers and transmitted to the sCMOS sensor. The sensor then discriminated the light source based on the distinct temporal activation sequence (Fig. 1D). Each full activation cycle lasted 200 ms, yielding a final sampling frequency of 5 Hz for the sCMOS-based system.

On the detection side, the optical signal collected by each receiving fiber undergoes bifurcation via a fiber-optic splitter, directing the light onto two separate regions of the sCMOS sensor. Each fiber terminates in a custom mounting bracket with integrated light shielding, ensuring direct coupling to the sensor (Fig. 1E). Two bandpass filters were placed in front of the sensor: a 400–780 nm filter to transmit light at ~695 nm, and a 760–1100 nm filter to transmit light at ~830 nm, both with >95% transmittance in their passbands.

The sCMOS sensor (IUA4200KME, Hangzhou Touptek Photonics Co., Ltd) measured 68 × 68 × 28.1 mm, weighed 270 g, and featured a 4.2-megapixel (2048 × 2048) array with a dynamic range of 68.1 dB. During each activation cycle, frame images were captured according to the predefined temporal sequence. Figure 1F shows the fully integrated hardware of the sCMOS-based fNIRS system.

### Data acquisition protocol for sCMOS-based fNIRS

**Fig. 2:**
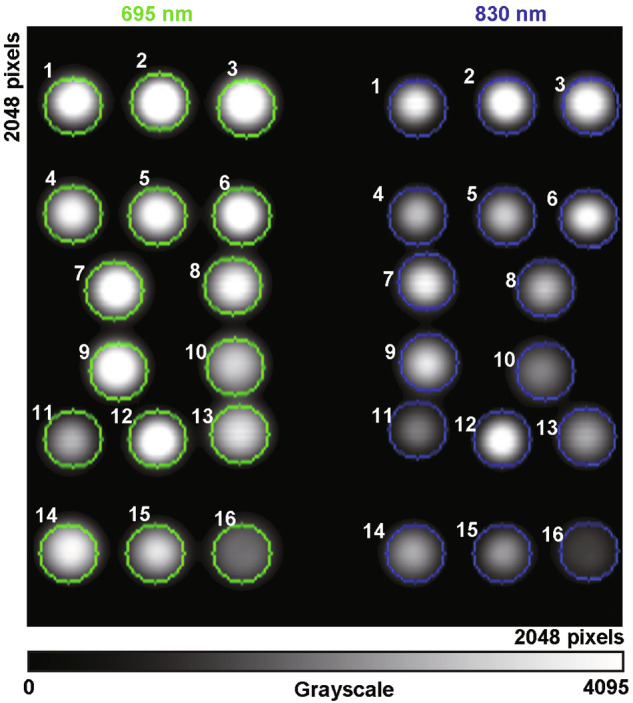
Dual-wavelength speckle imaging with the sCMOS sensor. One frame captures dual-wavelength (695/830 nm) speckle patterns emitted from the receiving fiber terminus.

As illustrated in Fig. 2, the sCMOS sensor captures time-series frame images in which each frame contains dual-wavelength circular speckle patterns emitted from fiber tips, with one half capturing 695 nm light and the other half capturing 830 nm light.

Circular regions of interest (ROIs) with a fixed 102-pixel radius were manually defined around user-selected reference points for image segmentation. Within these ROIs, the image data from the sCMOS sensor were converted into grayscale values using ToupView software (Hangzhou ToupTek Photonics Co., Ltd), followed by the calculation of mean grayscale values (*MGVs*).

### Signal quality and system performance assessment

High-fidelity signal acquisition is essential for fNIRS, as it enables precise chromophore separation and guarantees reproducible outcomes. Instrument performance was quantified through these primary metrics:

#### Signal-to-Noise Ratio (*SNR*)

**Fig. 3:**
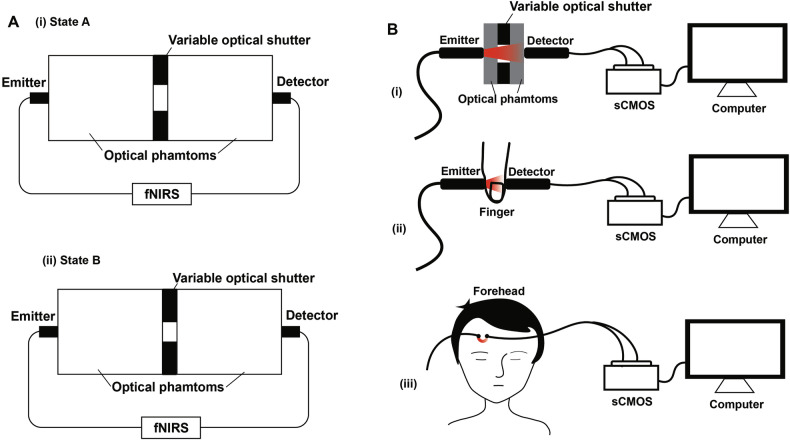
Test setup schematic for characterizing key sCMOS-based system performance metrics. **A** SNR evaluation. Experimental setup for SNR characterization per GB 9706.271–2022, using two optical phantoms and a variable optical shutter. A calibrated 3–4 dB perturbation was applied via aperture adjustment against a > 60 dB loss baseline to create (i) States A and (ii) B for differential measurement. **B** DOR validation. (i) Upper bound (${{MGV}}_{\max }$): Source power was increased using an optical phantom until reaching the non-saturating ${{MGV}}_{\max }$ of 4095 using an optical phantom. (ii, iii) In vivo validation: The defined upper bound was confirmed sufficient by avoiding saturation during (ii) finger transmission-mode and (iii) forehead scattering-mode (3 cm separation) measurements.

This metric quantifies the ratio of the target signal power to the background noise power. Characterization was performed in accordance with GB 9706.271–2022, employing two optical phantoms and a variable shutter (Fig. 3A). With a background optical loss of > 60 dB, a calibrated 3–4 dB perturbation was introduced by varying the aperture, creating distinct States A and B for differential measurement. SNR was calculated using:

1 $$SNR(dB)=20\times lo{g}_{10}\left(\frac{MG{V}_{A}-MG{V}_{B}}{\delta MGV}\right)$$

Where ${{MGV}}_{A}-{{MGV}}_{B}$ is the mean change in *MGV* upon transitioning from State A to State B, and $\delta {MGV}$ is the standard deviation of that difference. This procedure was repeated four times per channel.

#### Noise-equivalent power (*NEP*)

The *NEP* corresponds to an incident optical power when *SNR* = 1. Following the method reported by Bergonzi et al. for determining the per-pixel NEP (${{NEP}}_{{pix}}$) of the sCMOS sensor [14]:

2 $$NE{P}_{{pix}}(W/\sqrt{Hz})=\frac{{Qhc}}{\lambda t{\eta }_{\lambda }\sqrt{{BW}}}$$

Where *Q* is the read noise in electrons (a theoretical value of 3.57 e^-^ for the sCMOS sensor used), *h* is Planck’s constant, *c* is the speed of light (m/s), $\lambda$ is the operating wavelength (m), *t* is the exposure time (*t* = 0.025 s), ${\eta }_{\lambda }$ is the detector’s quantum efficiency (84.06% at 695 nm and 53.33% at 830 nm), and *BW* = 0.09 Hz is the bandwidth, corresponding to the final analysis bandwidth of 0.01–0.1 Hz.

Since the optical spot from a single fiber covers multiple pixels on the sensor (*N* ≈ 32686), the signals from these pixels are summed. The total *NEP* for the combined channel is then:

3 $$NE{P}_{{sum}}(W/\sqrt{Hz})\approx \sqrt{N}\times NE{P}_{{pix}}$$

The system’s actual *NEP* (${{NEP}}_{{act}}$) was determined directly under controlled weak‑light conditions where the detected signal amplitude equaled the system noise amplitude (*SNR* ≈ 1). The corresponding *MGV* (${{MGV}}_{{SNR}=1}$) was acquired with the sCMOS sensor operating in its high‑conversion‑gain mode. This value, expressed in analog‑to‑digital units (ADU), was converted to the total number of signal electrons $Q$ ${(Q}_{{SNR}=1})$ for the region of interest using the calibrated conversion gain (CG = 2.33 e⁻/ADU):

4 $${Q}_{{SNR}=1}=N\times MG{V}_{SNR=1}\times {CG}$$

The experimentally measured ${{NEP}}_{{act}}$ is then obtained by substituting ${Q}_{{SNR}=1}$ into the photometric relation:

5 $$NE{P}_{{act}}(W/\sqrt{Hz})=\frac{{Q}_{{SNR}=1}{hc}}{\lambda t{\eta }_{\lambda }\sqrt{{BW}}}$$

#### Dynamic optical range (*DOR*)

The *DOR* is defined as the ratio of the maximum nonsaturating to the minimum detectable optical power. It was characterized as follows. The upper bound (${{MGV}}_{\max }$) was first determined using an optical phantom (Fig. 3B(i)) by incrementally opening the variable optical shutter until the *MGV* of 4095 was reached. Subsequent in vivo measurements confirmed the validity of this upper limit, as no signal saturation was observed in either transmission-mode measurements on a human finger (Fig. 3B(ii)) or reflectance-mode measurements on the forehead with a 3 cm optode separation (Fig. 3B(iii)). The lower bound (${{MGV}}_{\min }$) was defined as the noise-equivalent gray value from the *NEP* characterization. The DOR was then calculated as:

6 $$DOR(dB)=10\times lo{g}_{10}\frac{MG{V}_{max}}{MG{V}_{min}}$$

#### Drift characterization

Long-term system stability was assessed by recording one hour of continuous optical signals from a silicone optical phantom (*μ*_*a*_ = 0.012 mm⁻¹, *μ*_*s*_*’* = 2.3 mm⁻¹ at *λ* = 695 nm) at a controlled ambient temperature of 25 °C. Drift was quantified using three metrics: (1) the linear drift rate (%/h), derived from a least-squares linear fit; (2) the Allan deviation, computed to identify the optimal averaging time and characterize noise types across time scales; and (3) the percentage of time within ± 3 standard deviations (σ), which indicates compliance with a stable operational envelope.

### APD-based fNIRS system

The APD-based fNIRS device (ETG ONE, 22 channels, Hitachi Medical Co.) was used for comparative analysis. To achieve light source identification, each light source was uniquely frequency-modulated at approximately 1.2 kHz. The device incorporates 8 emitters and 7 detectors, forming 22 channels, and operates at a sampling frequency of 10 Hz. Optical signals captured by the receiving fibers are transmitted to the APD, where they are converted into voltage signals. These signals are then amplified and undergo demodulation via dedicated circuitry based on their modulation frequencies. This process isolates the signal from each individual light source and recovers its positional information.

### Detection of scatterer-absorber phantom in vitro

#### Scatterer-absorber phantom design

The scatterer-absorber phantom (SA-phantom), containing Intralipid (scattering agent) and India ink (absorber), serves as a standard tissue-mimicking phantom for biomedical optical calibration [18]. Intralipid, a soybean oil-based emulsion, exhibits strong scattering and low absorption, effectively mimicking the scattering characteristics of biological tissue [19, 20]. India ink, a substance with low-scattering and high-absorption properties, simulates the absorption characteristics [21]. This SA-phantom can simulate the absorption and scattering properties of biological tissues based on different ratios of Intralipid and India ink. As research mentioned, during oxygen saturation (SO₂) variations, the primary alteration occurs in the tissue’s absorption characteristics [22]. Therefore, the SA-phantom maintains a fixed Intralipid concentration while progressively varying India ink concentrations to quantify the system’s absorption coefficient (*μ*_*a*_, mm⁻¹) measurement capability. The target *μ*_*a*_ ranges for the phantom were informed by the in vivo measurements of Bevilacqua et al. [23], Okada et al. [24] and Fukui et al. [25], who characterized *μ*_*a*_ and transport scattering (*μ*_*s*_*’*) coefficient (mm⁻¹) of adult and neonatal scalp, skull, gray matter, and white matter (see Table 1 for specific values). Based on this, we developed the SA-phantom to evaluate its optical response across a range of absorption strengths. The SA-phantom was prepared with predetermined optical properties (*μ*_*a*_ = 0.009 mm⁻¹, *μ*_*s*_*’* = 2.3 mm⁻¹ at *λ* = 695 nm; *μ*_*s*_*’* calculated per Staveren et al. [19]). Through stepwise addition of India ink (2 μL increments) into the SA-phantom, we achieved graded increases in *μₐ*.

**Table 1:** Optical properties of human brain tissue and the SA-phantom used for the validation measurements.

Reference & Tissue		*μ*_*a*_ mm^−1^	*μ*_*s*_*’* mm^−1^
Bevilacqua et al. [23] In vivo human brain	Skull	0.05 ± 0.02 (*λ* = 674 nm)	0.9 ± 0.1 (*λ* = 674 nm)
	Cortex (frontal lobe)	<0.02 ± 0.01 (*λ* = 674 nm)	1.0 ± 0.05 (*λ* = 674 nm)
	Cortex (temporal lobe)	0.02 ± 0.01 (*λ* = 674 nm)	1.0 ± 0.05 (*λ* = 674 nm)
	Cerebellar white matter	0.25 ± 0.05 (*λ* = 674 nm)	1.35 ± 0.1 (*λ* = 674 nm)
Okada et al. [24] Adult head	Scalp	0.018 (*λ* = 800 nm)	1.9 (*λ* = 800 nm)
	Skull	0.016 (*λ* = 800 nm)	1.6 (*λ* = 800 nm)
	Gray matter	0.036 (*λ* = 800 nm)	2.2 (*λ* = 800 nm)
	White matter	0.014 (*λ* = 800 nm)	9.1 (*λ* = 800 nm)
Fukui et al. [25] Neonatal head	Scalp	0.018 (*λ* = 800 nm)	1.9 (*λ* = 800 nm)
	Skull	0.016 (*λ* = 800 nm)	1.6 (*λ* = 800 nm)
	Gray matter	0.048 (*λ* = 800 nm)	0.5 (*λ* = 800 nm)
	White matter	0.037 (*λ* = 800 nm)	1.0 (*λ* = 800 nm)
SA-phantom	Baseline group	0.00899 - 0.01100 (*λ* = 695 nm)	2.3 (*λ* = 695 nm)
	Low-absorption group	0.01192 - 0.01254 (*λ* = 695 nm)	2.3 (*λ* = 695 nm)
	High-absorption group	0.03366 - 0.04639 (*λ* = 695 nm)	2.3 (*λ* = 695 nm)

In the context of functional brain activity, changes in tissue absorption are predominantly governed by hemoglobin dynamics. This relationship forms the foundational principle for quantitative fNIRS. Consequently, our sensitivity analysis focused on varying the absorption coefficient. Reported values for tissue *μ*_*a*_ in the literature exhibit considerable variation due to differences in experimental design and subject populations. To systematically evaluate the impact of tissue absorption properties on our measurements, we conducted simulations across three representative *μ*_*a*_ ranges (see Table 1):

**Baseline group** (*μ*_*a*_ = 0.00899–0.01100 mm^−1^, *μ*_*s*_*’* = 2.3 mm^−1^, *λ* = 695 nm): This range is anchored on the in vivo measurements of healthy human brain tissue reported by Bevilacqua et al. [23].

**Low-absorption group** (*μ*_*a*_ = 0.01192–0.01254 mm⁻¹, *μ*_*s*_*’* = 2.3 mm⁻¹ at *λ* = 695 nm): This range simulates conditions at the lower end of the measurements reported by Bevilacqua et al. [23] and Okada et al. [24].

**High-absorption group** (*μ*_*a*_ = 0.03366 – 0.04639 mm⁻¹, *μ*_*s*_*’* = 2.3 mm⁻¹ at *λ* = 695 nm): This range simulates conditions at the higher end of the measurements reported by Okada et al. [24] and Fukui et al. [25].

The *μ*_*s*_^*’*^ decreases with increasing wavelength in the NIR range. Based on reports for cerebral tissues [24] and Fukui et al. [25], the actual *μ*_*s*_^*’*^ at 695 nm is likely slightly higher than values reported at longer wavelengths. Therefore, for the purpose of this controlled sensitivity analysis, μs’ was held constant at 2.3 mm⁻¹ across all simulations.

#### Phantom measurement and validation

The optode arrays of the APD-based system (22 channels) and the sCMOS-based system (52 channels) were fixed on opposite sides of a large light-shielded container (approximately 80 × 40 × 60 cm). A black light-absorbing panel was securely positioned between the two arrays to prevent signal crosstalk. The distance between each array and the container’s inner wall was maintained at ≥ 5 cm. The container was filled with the SA-phantom, and a magnetic stirrer was placed beneath its center. Following each microtitration of India ink, the mixture was stirred for 5 min to ensure thorough homogenization. India ink was incrementally added until a stopping criterion was met: signal outputs across all channels stabilized, indicating that no further increase in *μ*_*a*_ could be detected with additional ink infusion.

Because the volume of India ink added each time was negligible, the resulting changes in total solution volume were not considered in the analysis. The *μ*_*a*_ of the phantom at each ink concentration was quantified using a spectrophotometer. Furthermore, variations in incident light intensity across emitters, attributable to differing degrees of fiber-optic wear, were measured and used to normalize the corresponding channel signals.

### Detection of blood-doped tissue-simulating phantom in vitro

Subsequently, we employed a blood-doped tissue-simulating phantom (BD-phantom) to validate the quantification accuracy of the sCMOS-based fNIRS system for dynamic changes in HbO and HbR concentrations.

A hermetically sealed, opaque vessel was filled with BD-phantom. Specifically, the formulation of the BD-phantom was based on the volume ratios and optical parameters of human tissue. In normal adults, cerebral blood volume is approximately 4–6 mL per 100 g of brain tissue [26]; thus, our BD-phantom contained 5% blood by volume. According to the method of Staveren et al. [20], we adjusted the Intralipid concentration to achieve a *μ*_*s*_*’* of 2.3 mm⁻¹, with saline comprising the remaining volume. The optode arrays of the APD-based system (22 channels) and sCMOS-based system (52 channels) were positioned on two opposing sides of the container, separated by a black light-absorbing panel to prevent crosstalk. A 5-cm minimum clearance was maintained between the arrays and container walls. The container was filled with BD-phantom, and a magnetic stirrer was centered beneath it. After oxygenating the BD-phantom to 100% SO_2_ with pure oxygen, we gradually added the oxygen-depleting agent sodium dithionite (S4340000, Shanghai Aladdin Biochemical Technology Co., Ltd.). Both systems continuously recorded ∆HbO and ∆HbR throughout the procedure. Concurrently, the BD-phantom was periodically sampled and analyzed as a reference using a blood-gas analyzer (BGA-102, Guangzhou Wondfo Biotech Co., Ltd.).

### Detection of fNIRS signal in vivo

This experiment aimed to concurrently acquire in vivo fNIRS signals using both sCMOS- and APD-based systems from the same subject during an identical Verbal Fluency Task (VFT) trial. All experimental protocols were reviewed and approved by the Institutional Review Board of West China Hospital, Sichuan University (Approval No. 20242537).

The sCMOS- and APD-based systems employ distinct optical acquisition methodologies. The sCMOS-based system operates on a time-division multiplexed continuous-wave (TDM-CW) principle, demultiplexing signals based on their temporal sequences. In contrast, the APD-based system utilizes frequency-encoded continuous-wave (CW) light, which is resolved via lock-in amplification with frequency demodulation.

A critical consideration in the experimental design was the selection of the light source. CW light sources from the APD-based system were specifically utilized, primarily due to the inability of the sCMOS-based system’s TDM-CW sources to provide the frequency encoding capability necessary for signal identification and demodulation by the APD-based system. This approach, however, introduced challenges for the sCMOS-based system, which cannot discriminate multiple CW sources. To resolve this, we ensured that only a single source was active—a condition under which the sCMOS-based system functions accurately. Consequently, two APD sources were positioned sufficiently distant from each other over the left and right frontal lobes, thus satisfying the operational requirements of both systems while minimizing interhemispheric crosstalk.

**Fig. 4:**
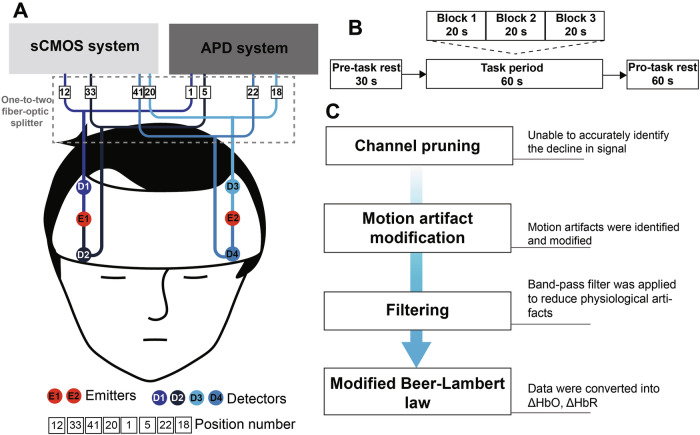
In vivo fNIRS signal acquisition scheme. **A** Diagram illustrating how both systems acquire signals from the same cortical regions during a single Verbal Fluency Task (VFT) trial. Two emitters (E1, E2) were positioned over the left and right prefrontal cortex. Each emitter was paired with two detectors (E1 with D1 and D2; E2 with D3 and D4). The optical fiber from each detector was connected to a 1 × 2 fiber-optic splitter (dashed grey box), which routed identical light signals concurrently to both the sCMOS- and APD-based systems for simultaneous recording. **B** Standardized VFT protocols. **C** The fNIRS data preprocessing pipeline.

The setup for simultaneous data acquisition is schematized in Fig. 4A. Two optical emitters from the APD-based system were positioned over the left and right frontal lobe, respectively. Two detectors were placed adjacent to each emitter to capture the back-scattered light. To facilitate concurrent signal acquisition from an identical cerebral event, the optical fiber from each detector was connected to a 1 × 2 fiber-optic splitter. This configuration passively divided the collected light signal from each detection point, directing one output to the sCMOS-based system and the other to the APD-based system. Thus, during a single VFT trial, both systems acquired data synchronously from identical cortical regions and time points, enabling a direct comparative analysis of their respective signals.

Standard VFT protocols were administered to five informed, consenting healthy subjects monitored concurrently by the APD-based and sCMOS-based systems (4 channels/subject, 20 total). Consistent with established VFT implementations, the paradigm comprised three sequential epochs (Fig. 4B) [27–29]:

- A 30 s pre-task rest (paced counting, 1 - 5);
- A 60 s task period (semantically valid Mandarin word generation cued by logographic stimuli, e.g., shàng, shí, shuō, and jiā);
- A 60 s post-task rest period (resting state).

Recordings of APD-derived optical intensities (*I*) and sCMOS sensor *MGVs* were obtained across all channels for further analysis.

The main preprocessing steps in the fNIRS data analysis included (Fig. 4C) [30]: (1) Channel exclusion: During the SA-phantom experiment, channels showing no reduction in fNIRS readings at both wavelengths during India ink titration were identified as non-responsive and pruned; (2) Motion artifact correction: Signal segments exceeding predefined thresholds for either standard deviation (STDthresh = 10) or amplitude (AMPthresh = 5) were classified as artifactual. A 0.5-s time window (tMask) around each artifact was marked as a motion artifact mask. Channels were rejected if >5% of their time points were contaminated by motion artifacts. Furthermore, if >30% of channels within a session were marked as bad in at least one participant, the corresponding channel‑pair data were excluded from analysis [31, 32]. (3) Physiological artifacts removal: artifacts from respiratory cycles and cardiac pulsations were attenuated using a Butterworth band-pass filter (0.01–0.2 Hz cutoff frequency) [29]; (4) Hemodynamic conversion: filtered data were processed via the modified Beer–Lambert law to derive ∆HbO and ∆HbR [33].

### Statistical analysis

fNIRS data were processed and analyzed using MATLAB R2023a (The MathWorks, Inc., Natick, MA, USA). The accuracy of both systems in responding to *μ*_*a*_ and SO_2_ was assessed via linear regression, with the coefficient of determination (*R*^*2*^) representing the proportion of variance explained by the model; an *R*^*2*^ value closer to 1 indicates a better fit. To compare fNIRS signals between the sCMOS- and APD-based systems, we performed correlation analysis. Pearson correlation was used for normally distributed data; otherwise, Spearman correlation was applied. A *P*-value of less than 0.05 was considered statistically significant.

## Results

### System performance specifications and validation

The key technical specifications of the custom-built sCMOS-based system are summarized in Table 2. Comprehensive characterization confirmed that the system performance meets the requirements for high-quality functional brain imaging.

**Table 2:** Technical Specifications of the sCMOS-based system.

Category	Parameter		Specification (Performance)
Physical & Optical Properties	Light source		LEDs, 695 nm (n = 17) / 830 nm (n = 17)
	Detector		Scientific Complementary Metal-Oxide-Semiconductor (sCMOS) sensor
	Source-Detector Separation (SDS)		30 mm
	Optoelectronic Unit Dimensions		68 × 68 × 28.1 mm
	Optoelectronic Unit Weight		270 g
Signal Characteristics	Signal-to-Noise Ratio (*SNR*)		> 50 dB
	Noise-equivalent power (*NEP*)	Theoretical value	695 nm: 5.29 pW/√Hz 830 nm: 6.96 pW/√Hz
		Actual value	695 nm: 9.91 ± 0.14 pW/√Hz 830 nm: 13.43 ± 0.19 pW/√Hz
	Dynamic Optical Range (*DOR*)		695 nm: 33.40 dB 830 nm: 33.29 dB
Signal Stability	Linear drift rate		< 0.6% per hour
	Stability (Allan Deviation)		695 nm: 0.15 ± 0.16 830 nm: 0.10 ± 0.09
	Time within ± 3σ		695 nm: 99.59 ± 0.66% 830 nm: 99.80 ± 0.57%

σ denotes the standard deviation.

*SNR* was evaluated across all 52 channels in accordance with GB 9706.271–2022. As shown in Supplementary Fig. 1, the SNR exceeded 50 dB for all channels, with mean values of 52.31 ± 1.34 dB at 695 nm and 50.77 ± 0.72 dB at 830 nm.

Theoretically, the *NEP* was calculated to be 5.29 pW/√Hz at 695 nm and 6.96 pW/√Hz at 830 nm. Experimentally, the measured *NEP* values were 9.91 ± 0.14 pW/√Hz and 13.43 ± 0.19 pW/√Hz at 695 nm and 830 nm, respectively (Supplementary Fig. 2).

The *DOR* was derived from the maximum and minimum measurable *MGV*. With a system ${{MGV}}_{\max }$ of 4095 and measured ${{MGV}}_{\min }$ values of 1.87 (695 nm) and 1.92 (830 nm), the *DOR* was calculated to be 33.40 dB and 33.29 dB for 695 nm and 830 nm, respectively. To validate the *DOR* under realistic conditions, we performed measurements through a finger (to simulate tissue attenuation) and across the forehead (to simulate a typical imaging scenario). The resultant ${{MGV}}_{\max }$ values under these conditions were 1015.52 (695 nm, finger), 989.06 (830 nm, finger), 335.29 (695 nm, forehead), and 326.56 (830 nm, forehead)—all well below the system’s full-scale saturation limit of 4095. These results confirm that the system retains sufficient dynamic range to accommodate the significant signal attenuation inherent to in vivo tissue measurements.

Signal stability was assessed by continuous measurement using a silicone phantom immediately after system startup. All channels exhibited a linear drift rate of less than 0.6%/h (Supplementary Fig. 3A). The Allan Deviation, which quantifies long-term stability, was 0.15 ± 0.16 at 695 nm and 0.10 ± 0.09 at 830 nm (Supplementary Fig. 3B). Furthermore, the raw intensity signal remained within ± 3σ of its mean value for 99.59 ± 0.66% (695 nm) and 99.80 ± 0.57% (830 nm) of the total measurement time (Supplementary Fig. 3C), demonstrating excellent short-term stability and noise performance.

While these stability metrics, obtained without a dedicated warm-up period, indicate a robust system performance, we recommend a minimum preheating duration of 5 min prior to experimental data collection. This recommendation is based on the fundamental principle that the center wavelength of LED emission is intrinsically sensitive to junction temperature. A controlled warm-up period facilitates thermal equilibrium within the optoelectronic modules, thereby minimizing potential wavelength drift and ensuring optimal long-term output power stability.

### Sensitometric characteristics of sCMOS-based system in SA-phantom

**Fig. 5:**
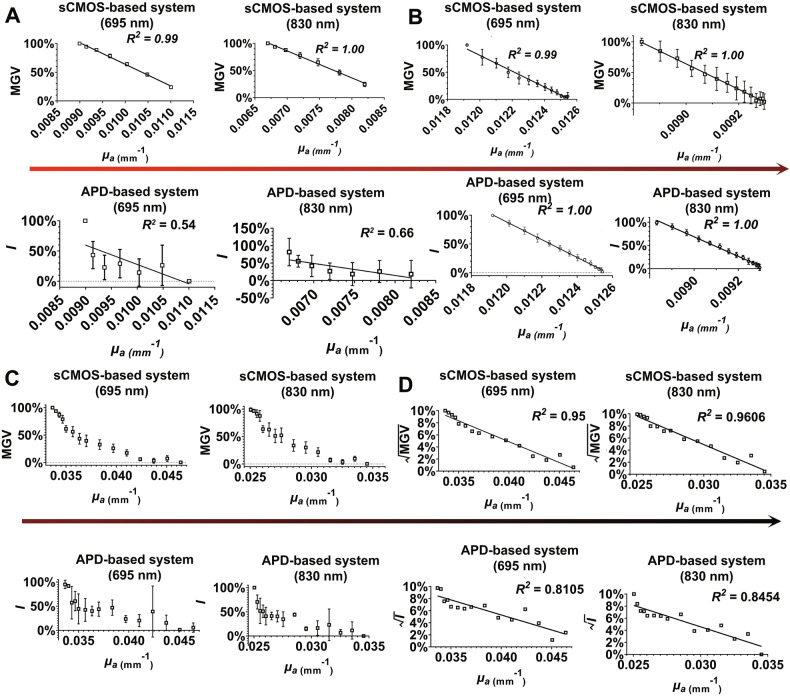
SA-phantom comparison of sCMOS- vs. APD-based system. SA-phantom (*μₐ* = 0.009 mm⁻¹ at *λ* = 695 nm) underwent ink titration until signal degradation. Signal consistency was evaluated in both systems across *μₐ* ranges: (**A**) Baseline group (*μₐ* 0.009–0.011 mm⁻¹ at *λ* = 690 nm). **B** Low-absorption group (*μₐ* 0.0119 − 0.01255 mm⁻¹ at *λ* = 695 nm). **C** High-absorption group (*μₐ* 0.0336–0.0464 mm⁻¹ at *λ* = 690 nm). **D** Square root transformed signal consistency between sCMOS- and APD-based systems in the high-absorption group (*μₐ* 0.0336–0.0464 mm⁻¹ at *λ* = 695 nm).

Initially, the SA-phantom exhibited low *μ*_*a*_ (*μ*_*a*_ = 0.009 mm⁻¹, *μ*_*s*_*’* = 2.3 mm⁻¹ at *λ* = 695 nm). India ink was titrated into the phantom. After acquiring and normalizing light intensities across all channels, we found that in the baseline group (*μ*_*a*_ 0.009–0.0110 mm⁻¹ at *λ* = 695 nm), the sCMOS-based system showed higher sensitivity to *μ*_*a*_ changes. This was evidenced by linear correlations (*R*^*2*^ ≥ 0.99) between the *μ*_*a*_ and the MGV variation (%) at both 695 nm and 830 nm. In contrast, the APD-based system exhibited limited sensitivity in detecting *μ*_*a*_ changes, as indicated by weak linear regression fits (*R*^*2*^ = 0.54 at 695 nm; *R*^*2*^ = 0.66 at 830 nm) (Fig. 5A).

We proceeded to modify the ink concentration in the SA-phantom, starting with *μ*_*a*_ = 0.0119 mm⁻¹ (*μ*_*s*_^*’*^ = 2.3 mm⁻¹ at *λ* = 695 nm) in the low-absorption group. India ink was incrementally introduced into the SA-phantom. Results showed that both the sCMOS- and APD-based systems effectively detected *μ*_*a*_ changes, with all *R*^*2*^ values > 0.99 (Fig. 5B).

Next, we evaluated both systems’ ability to detect *μ*_*a*_ variations at elevated *μ*_*a*_ (*μ*_*a*_ = 0.034 mm^−1^, *μ*_*s*_^*’*^ = 2.3 mm^−1^ at *λ* = 695 nm) in the high-absorption group. At these higher *μ*_*a*_, the signal variation deviated from linearity (Fig. 5C). To address signal attenuation and increased noise in the low-signal regime, we applied a square-root transformation to the signal. This transformation stabilizes the variance of Poisson-distributed noise, where the standard deviation scales with the square root of signal intensity [34]. As shown in Fig. 5D, the transformed data exhibited a monotonic decrease in both systems’ readings with increasing *μ*_*a*_—though with weaker linearity than previously observed. The sCMOS-based system maintained linear correlations (*R*^*2*^ > 0.95), while the APD-based system showed reduced performance (*R*^*2*^ = 0.8105 and 0.8454 at 695 nm and 830 nm, respectively). At this *μ*_*a*_ (0.046 mm^−1^ at 695 nm), subsequent titration of India ink did not produce further signal attenuation in either fNIRS system.

### Sensitometric characteristics of sCMOS-based system in BD-phantom

The BD-phantom enables controlled assessment of system accuracy in quantifying ΔHbO under defined SO_2_ conditions. Initially, the SO_2_ was 100%, as confirmed by blood-gas analysis. Gradual titration of the oxygen-consuming agent induced SO_2_ decrease.

**Fig. 6:**
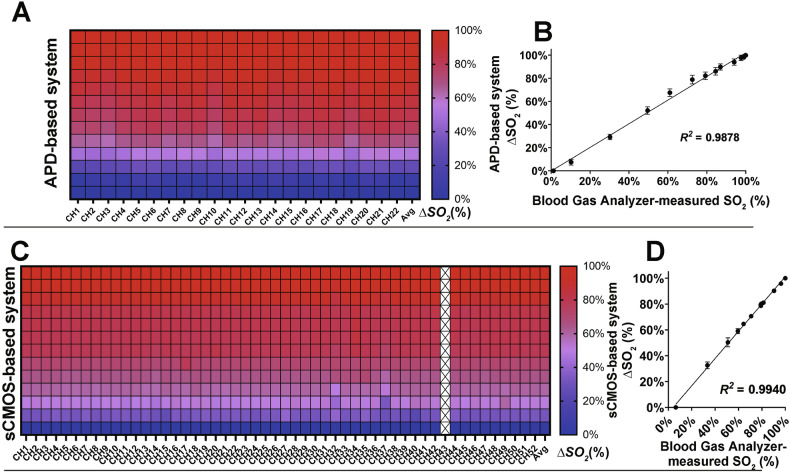
Performance evaluation of APD- and sCMOS-based systems in BD-phantom detection. **A** ΔSO_2_ mapping using the APD-based system. **B** APD-based system: ΔSO_2_ vs. measured SO₂ regression. **C** ΔSO_2_ mapping using the sCMOS-based system (52 channels). **D** sCMOS-based system: ΔSO_2_ vs. measured SO₂ regression.

Both systems effectively tracked the dynamics of SO_2_ in the BD-phantom. In the APD-based system, all channels exhibited strong performance in SO_2_ detection, as illustrated in the heatmap (Fig. 6A), where color intensity corresponds to SO_2_ levels (red indicating higher values). The measured ΔSO_2_ showed a linear correlation with the reference SO_2_ values (*R*^*2*^ = 0.9878, Fig. 6B). For the sCMOS-based system, as depicted in Fig. 6C, with the exception of CH43, which was excluded because artifacts accounted for >5% of its total signal, the remaining channels accurately detected SO_2_ changes, yielding *R*^*2*^ = 0.9940 (Fig. 6D).

### Performance of the sCMOS-based system during the VFT task

To enable a direct comparison of signals from the two fNIRS systems, we implemented a bifurcated fiber design at each detector. The raw signals from both systems were processed independently using identical algorithms, thereby eliminating potential discrepancies arising from differences in system software.

**Fig. 7:**
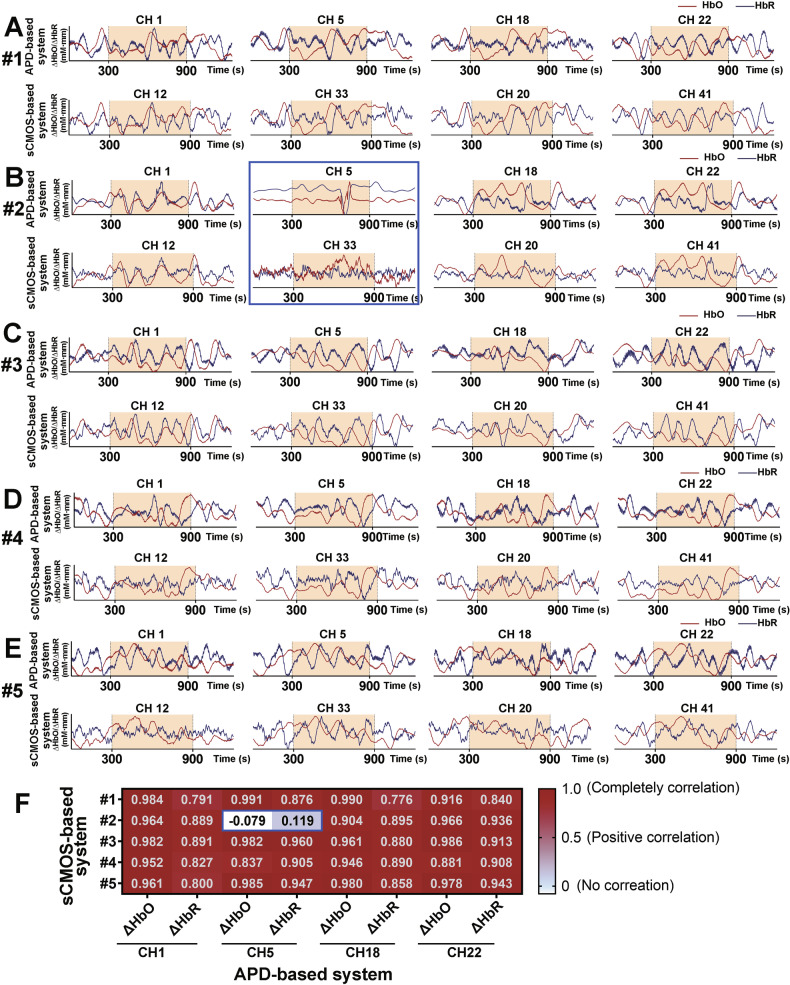
VFT task results and inter-system consistency. **A-E** ΔHbO (red) and ΔHbR (blue) for volunteers #1-#5. **F** Correlation heatmap (*Rs* values) between the two systems. Blue boxes highlight channels with low inter-system correlation.

Results for subjects #1–5 (Fig. 7A-E) show the following paired channels: APD-based system-CH1/sCMOS-based system-CH12, CH5/CH33, CH18/CH20, CH22/CH41. ΔHbO and ΔHbR waveforms from the two systems showed strong concordance. Nevertheless, Subject #2 (Fig. 7B, blue box) displayed anomalous behavior in one channel pair: the waveforms from the two systems were mutually inconsistent and diverged from those of other channels, which was attributed to physical displacement of the detection fiber.

Spearman correlation analysis, implemented due to non-normally distributed data, revealed robust channel-pair correlations (*Rs* > 0.77) across most measurements (Fig. 7F). The maximum observed correlation was *Rs* = 0.991 (ΔHbO, Subject #1: CH5/CH33), while the minimum valid correlation within normally functioning channels was *Rs* = 0.776 (ΔHbR, Subject #1: CH18/CH20). However, the outlier channel pair marked in blue (#2: APD-CH5/sCMOS-based system-CH33) exhibited near-zero correlations (*Rs* = −0.079 for ΔHbO and 0.119 for ΔHbR).

## Discussion

Over time, semiconductor photodetectors have migrated from the realm of scientific research into the medical field and daily life. This technological evolution has encompassed charge coupled device (CCD)—including full-frame CCDs (FFCCDs) and electron-multiplying CCDs (EMCCDs)—as well as complementary metal-oxide-semiconductor (CMOS) sensors and their scientific-grade counterparts, scientific CMOS (sCMOS) sensors. Continuous innovations have yielded larger photosensitive areas, lower noise floors, wider dynamic ranges, more compact form factors, simplified architectures, and improved thermal management.

The growing global burden of psychiatric disorders necessitates diagnostic approaches beyond conventional symptom scales. fNIRS addresses this need by quantifying cortical hemodynamic activity through ΔHbO and ΔHbR. However, conventional benchtop fNIRS systems—constrained by their APD-based detection architecture—face fundamental limitations in miniaturization, which increasingly conflict with contemporary demands for both research and clinical applications.

Beyond CW technology, multiple advanced fNIRS modalities, including frequency-domain (FD) [35] and time-domain (TD) technologies, have demonstrated superior potential for high-precision measurement and quantitative parameter estimation by overcoming inherent limitations of conventional CW-fNIRS [36]. FD systems employ modulated light sources to simultaneously resolve *μ*_*a*_ and *μ*_*s*_^*’*^, enabling absolute quantification of hemodynamic parameters [37]. In contrast, TD systems utilize ultrashort laser pulses to derive tissue optical properties from photon time-of-flight distributions, achieving enhanced depth penetration and unparalleled accuracy in deep-tissue interrogation [38]. Despite these advantages, CW-based systems remain the most widely adopted fNIRS technology in clinical settings due to their operational simplicity and cost-effectiveness [39].

Within CW-fNIRS devices, PDs and APDs dominate current devices. PDs, with their compact form factor, are employed in portable systems like NIRx Medical Technologies’ NIRScout and NIRSport2, Spectratech Inc.‘s OEG series, and Kernel’s Flow. In contrast, APDs represent prevalent detectors in fNIRS systems [36], though their larger size necessitates additional avalanche circuits and phase-locked loops, as exemplified by Shimadzu Corporation’s LABNIRS and Hitachi’s ETG series.

In theory, the integration of sCMOS sensors into the fNIRS system could significantly reduce device complexity while improving portability. As demonstrated in Fig. 1F, the sCMOS-based system is smaller in size than conventional benchtop fNIRS devices. While future work will pursue further miniaturization, the present study primarily investigates the integration of sCMOS sensors for fNIRS applications and evaluates their capability to replace traditional photodetectors in measuring ΔHbO. To this end, we developed an sCMOS-based fNIRS platform and implemented a comprehensive experimental validation framework.

In summary, the custom sCMOS-based fNIRS system demonstrates excellent performance across core optical parameters essential for fNIRS. Key metrics—including a high *SNR* ( > 50 dB), low *NEP* ( ~ 10 pW/√Hz), sufficient *DOR* ( > 33 dB), and outstanding temporal stability (drift < 0.6% /h, Allan Deviation ~0.1–0.15, and >99.5% time within ± 3σ)—collectively exceed typical system requirements.

Our optical phantom experiments demonstrated the sCMOS-based system’s superior detection accuracy over APD-based systems across both strong and weak light conditions. Under strong light, APD detectors exhibited pronounced susceptibility to saturation effects, whereas the sCMOS sensor maintained detectable photonic signal acquisition. Conversely, in weak light, the APD-based system failed to match the performance of our 68.1-dB dynamic range sCMOS detector.

This performance difference can be explained by two intrinsic limitations of the APD-based system: (1) nonlinear photoelectric response characteristics of APDs in weak-signal regimes; and (2) proprietary signal processing algorithms inherent to its architecture. As a closed-architecture commercial device (not an open-engineering platform), the APD-based fNIRS system denies access to raw photoconversion outputs—despite extensive attempts to interface directly with the detector, its proprietary encapsulation blocks signal interception. Consequently, researchers can only obtain manufacturer-processed signal values. Under severe attenuation, signal optimization protocols in the firmware systematically flag most channels as invalid. This appears in the real-time interface as predominantly disabled channels showing signal stasis during ink titration.

Under weak scattering conditions, this profound photonic attenuation aligns with the meta-analysis by Kwasa et al. [40], which examined demographic reporting practices and phenotypic considerations in fNIRS studies across leading biomedical optics and human neuroscience journals (2007–2022). Their analysis identified a critical methodological gap: existing fNIRS hardware architectures and analytical pipelines commonly fail to account for melanin-mediated NIR photon attenuation. Specifically, they proposed that elevated melanin concentration induces systematic nonlinear signal attenuation—a physiologically significant confounder that remains poorly characterized in fNIRS photobiology. The authors urge an urgent paradigm shift: the field must transform its technological frameworks and experimental practices to develop inclusive fNIRS methodologies. Such advancements must explicitly address melanin’s spectral interference while ensuring measurement validity across diverse phenotypic characteristics, including (but not limited to) melanin density, hair morphology, and skin reflectance properties.

The sCMOS-based system’s output optical fiber produces a light spot, and by integrating its *MGVs*, it enhances weak-light detection, thereby unexpectedly mitigating the low-light limitations of the sCMOS sensor.

Regarding validation methods, blood phantoms represent a standard and effective approach for evaluating fNIRS accuracy in measuring ΔHbO. The complete optode arrays from both systems were immersed in a BD-phantom within a light-proof container, enabling simultaneous multi-channel measurements. The results confirmed both systems’ exceptional accuracy in measuring ΔHbO, showing excellent consistency with blood gas analyzer measurements across 0–100% SO₂ ranges. While clinically relevant SO_2_ variations typically occur within a narrower range (60–100%) [41], this comprehensive validation establishes both systems as metrologically validated tools for precise SO_2_ quantification across the full physiological spectrum.

For adequate signal acquisition, sCMOS sensors require sufficient exposure time. In our configuration, a sampling rate of 5 Hz was achieved by balancing the light source activation frequency with the sensor exposure time, which involved sequentially sampling the four detector groups. Although currently below mainstream fNIRS standards, we are addressing this limitation through hardware upgrades and parallel sCMOS sensor array implementation in ongoing research.

Our findings demonstrate the sCMOS-based fNIRS’s non-inferiority to conventional APD-based systems. Through optimized fiber design, the sCMOS-based system achieved comparable performance to APD within identical brain regions, with Rs ranging from 0.8–0.991.

## Conclusion

We developed an fNIRS system that employs an sCMOS sensor as the optical detector, representing a transformative approach in neuroimaging with potential for future psychiatric applications. This innovative architecture replaces traditional APD-based systems, which require multiple discrete APDs paired with lock-in amplifiers. Remarkably, a single sCMOS focal-plane array can substitute for dozens of individual APD units, simultaneously eliminating complex control circuitry and achieving a significantly more compact form factor than conventional benchtop fNIRS systems. Through comprehensive validation involving phantom studies (including both scatterer-absorber and blood-doped tissue-simulating phantoms) and in vivo measurements in human volunteers, we demonstrated performance comparable to established APD-based fNIRS technologies, thereby providing evidence for the non-inferiority of this sCMOS-based approach.

## Supplementary information

Suplymentary materials

## Data Availability

Data described in this manuscript are available from the corresponding author upon reasonable request.
