# Supplementary material for: sCMOS-based fNIRS system: validation via optical performance and cortical response
Source: Transl Psychiatry. 2026 Apr 9;16:260. doi: 10.1038/s41398-026-03992-w (PMC13183969; doi:10.1038/s41398-026-03992-w)
Supplement: Supplementary file 1 — Suplymentary materials [file 41398_2026_3992_MOESM1_ESM.docx]

**Suplymentary materials**


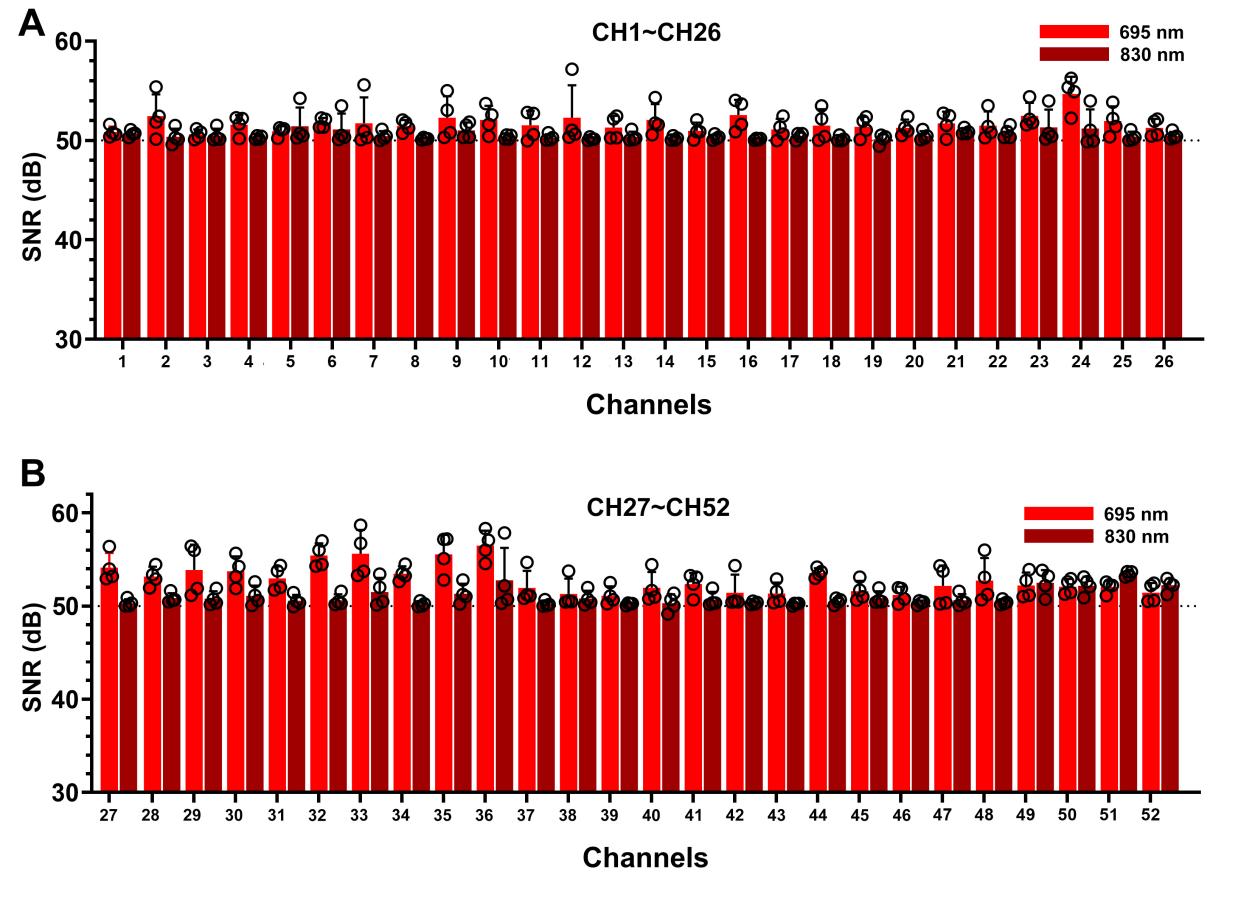


**Supplementary Figure 1**. **Measured SNR across all 52 channels of the sCMOS-based system.** (**A**) SNR for channels 1–26. (B) SNR for channels 27–52. Data points and error bars represent the mean ± standard deviation from four replicate measurements per channel.


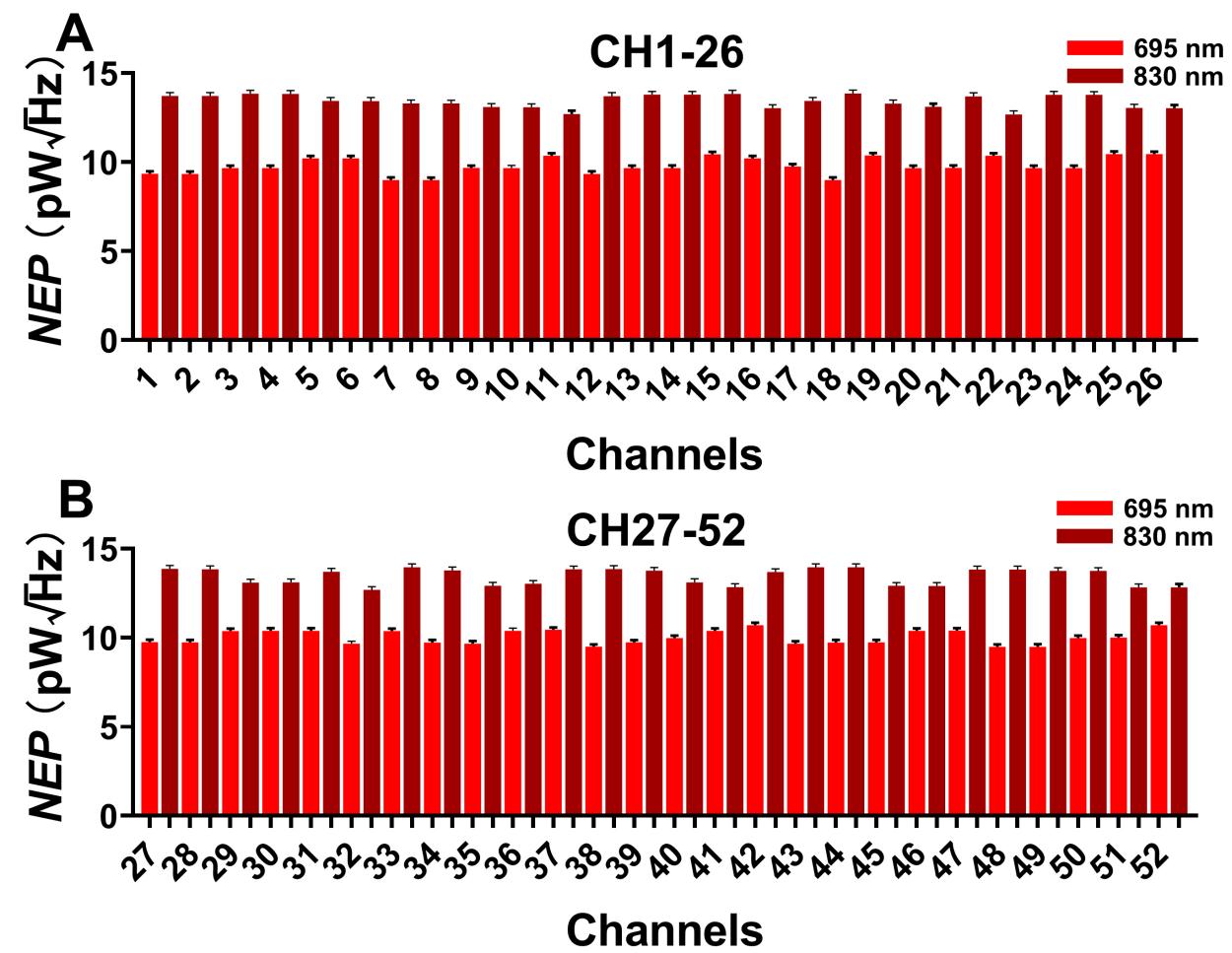


**Supplementary Figure 2. NEP across all 52 channels of the sCMOS-based fNIRS system.** (**A**) NEP for channels 1-26. (**B**) NEP for channels 27-52. Bar heights and error bars represent the mean ± standard deviation from four replicate measurements per channel.


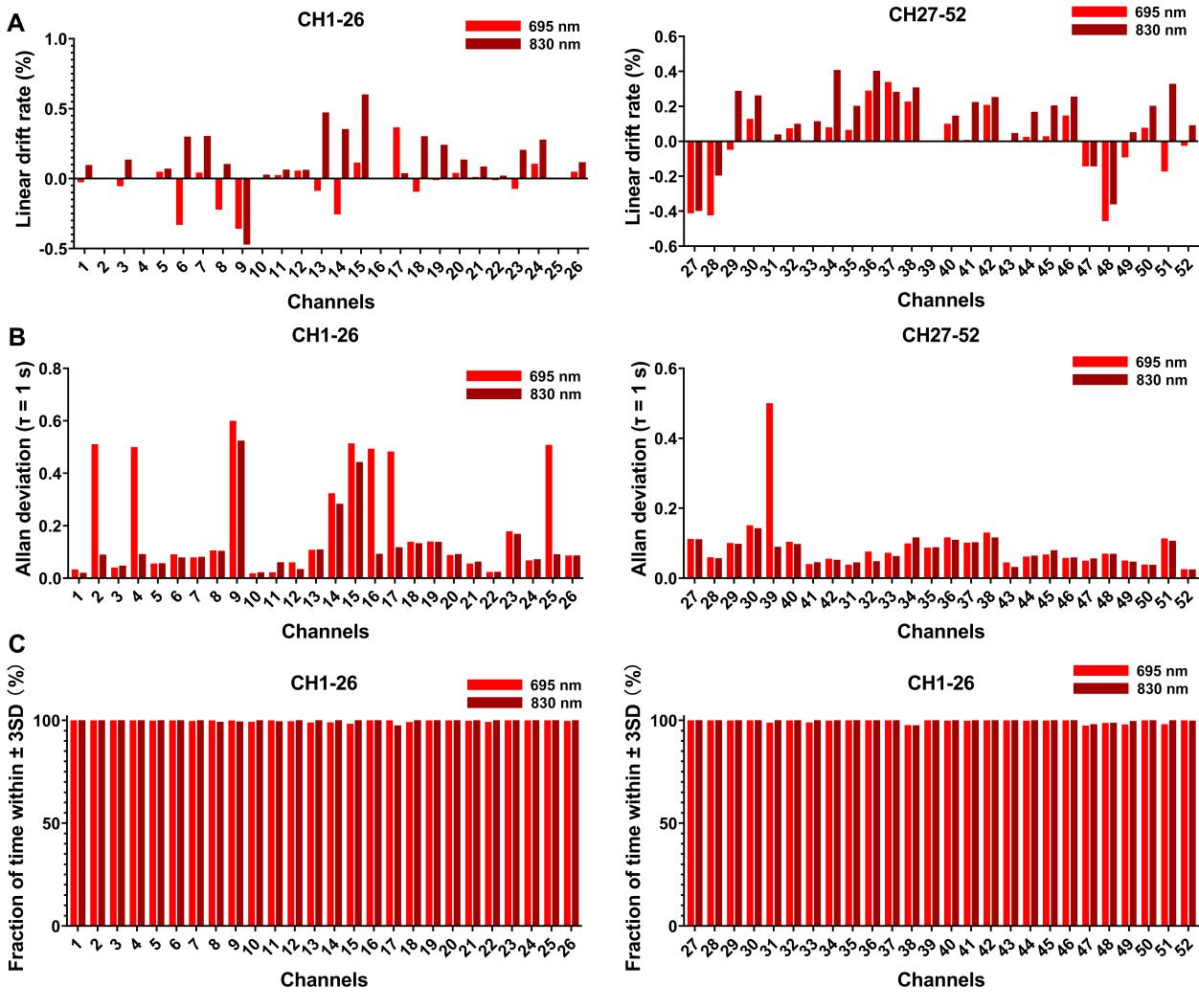


**Supplementary Figure 3. Assessment of long-term system stability.** (**A**) Linear drift rate per hour for all channels. (**B**) Allan Deviation for all channels. (**C**) Percentage of time the intensity signal remained within ±3 standard deviations (±3SD) of its mean for all channels.
